# Metagenomic data on bacterial diversity profiling of Arabian sea sediment by amplicon sequencing

**DOI:** 10.1016/j.dib.2019.104791

**Published:** 2019-11-11

**Authors:** Harisree P. Nair, Sarita G. Bhat

**Affiliations:** Department of Biotechnology, Cochin University of Science and Technology, Kochi, 682022, Kerala, India

**Keywords:** Community genome, Arabian sea, 16S rRNA, Marine

## Abstract

This data is about the microbial community genome analysis of Arabian sea sediment by Illumina sequencing by targeting the hypervariable region V3 of 16S rRNA gene. The data analysis revealed the existence of numerous unknown sequences, indicating a large unexploited bacterial diversity in the area. The raw sequence data used for analysis is available in NCBI under the Sequence Read Archive (SRA) with the BioProject No. PRJNA397165 and SRA accession number SRP125840.

**Table:** Specifications Table

Subject area	*Biology*
More specific subject area	*Marine Metagenomics*
Type of data	*Figures*
How data was acquired	*Illumina MiSeq platform*
Data format	*Raw and analyzed*
Experimental factors	*Arabian Sea sediment of 96 m depth were collected, representing the most productive epipelagic zone*
Experimental features	*Metagenomic DNA extraction and amplicon sequencing of V3 region of 16S rRNA gene*
Data source location	*Arabian Sea (9°59′10.9968″ N; 75° 39′ 26.4564″ E)*
Data accessibility	*The sequencing data is available in NCBI under the Sequence Read Archive (SRA) with the BioProject No. PRJNA397165 and SRA accession number SRP125840. The direct URL to data is*https://www.ncbi.nlm.nih.gov/sra/?term=SRP125840

**Table:** 

**Value of the Data** • The data provides insights into the hidden microbial diversity of Arabian sea sediments which can utilized as a treasure trove of novel biomolecules • The sequencing data is publicly available for comparative studies of microbial diversity in global oceans. • The scientific community is informed through the study about the existence of several unidentified sequences indicating the high incidence of novel yet-to-be cultured bacteria in the Arabian sea epipelagic sediments

## 1 Data

The largest habitable space for living organisms, particularly microorganisms is the marine realm, covering 70% of the planet surface. These microbial communities are key players in marine ecosystem maintenance [1]. Study of marine microbial biodiversity is of great significance, for it enables understanding biogeochemical cycles prevailing in the area. To harness these enormous genetic diversities in toto, metagenomic procedures can be applied. However, advances in next-generation sequencing methods have accelerated the large-scale exploration of taxonomic diversity of bacterial communities from diverse environments [[2], [3], [4]]. The complete focus is on presenting the taxonomic profile of bacterial communities of Arabian sea sediment.

**Fig. 1:**
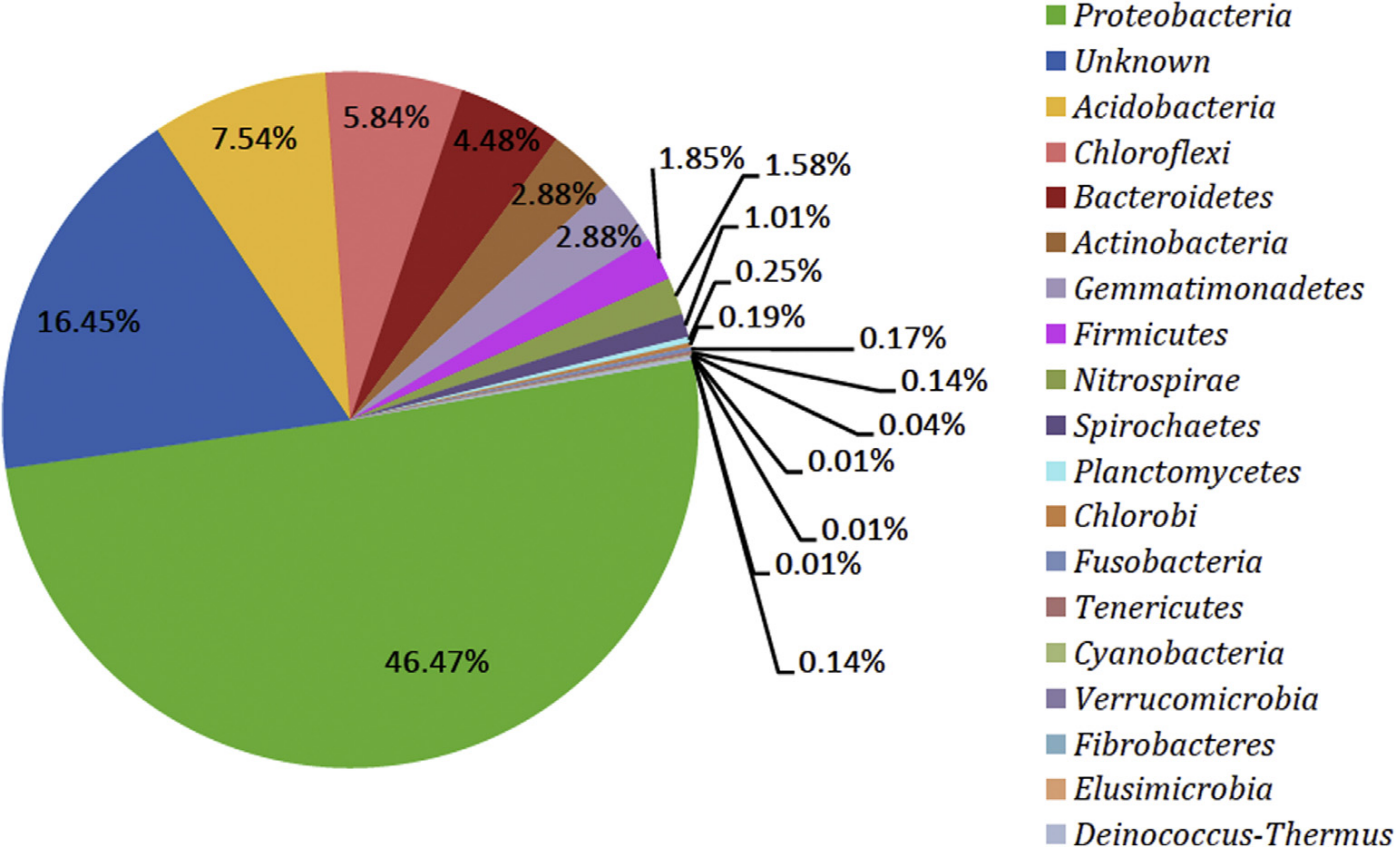
Taxonomic distribution of OTUs at phylum level from Arabian sea sediment.

**Fig. 2:**
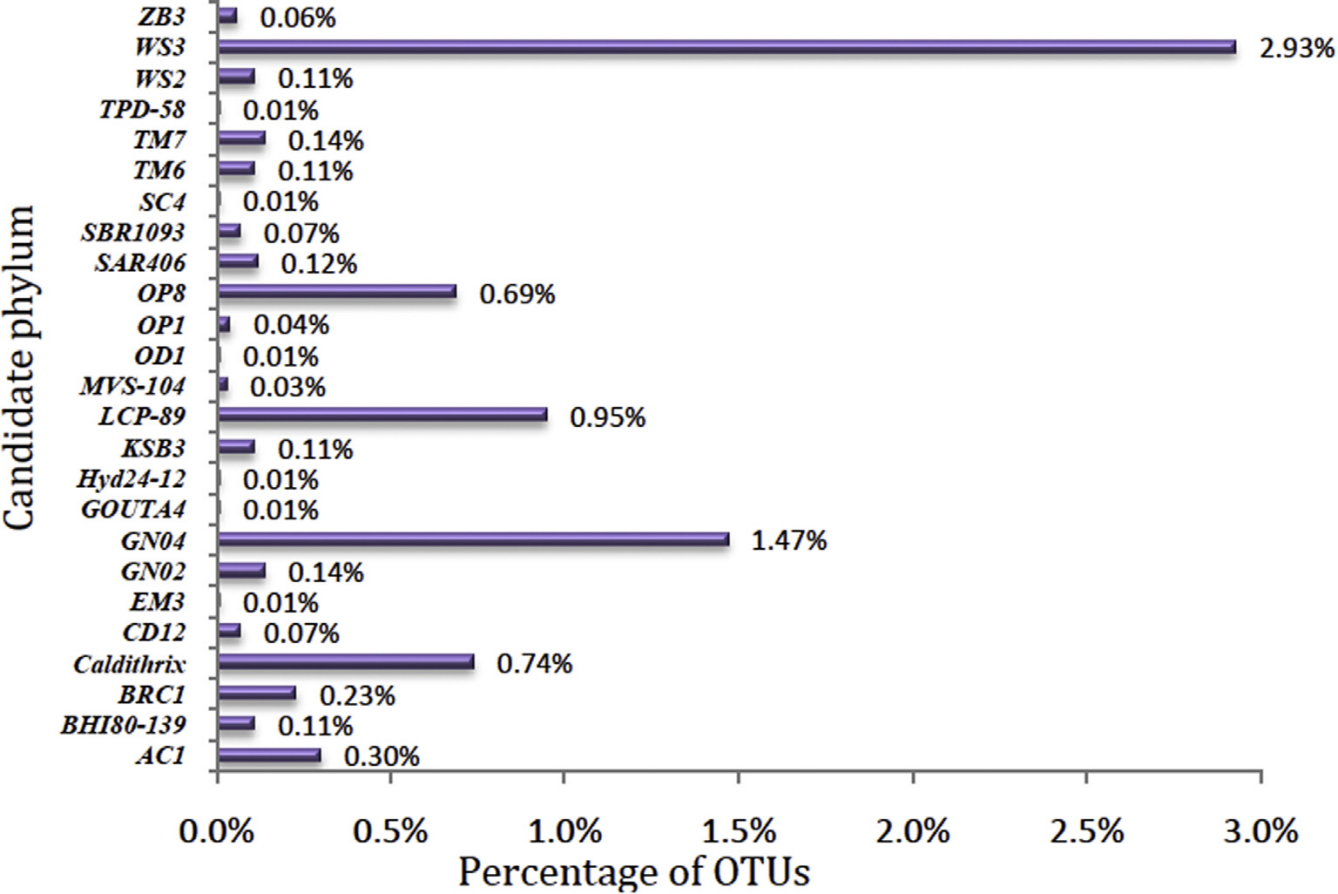
Taxonomic classification of OTUs at candidate phylum level from Arabian sea sediment.

6309 Operational Taxonomic Units (OTUs) were identified from the sequencing data and segregated into diverse taxonomic level of bacterial domains, which were classified into 43 bacterial phyla including 18 formally described bacterial phyla (Fig. 1) and 25 candidate phyla (Fig. 2).

Phylum *Proteobacteria* with 2932 OTUs was most abundant (at 46.47% of the total diversity). 16.45% (1038 OTUs) represented novel yet to be cultured organisms in Arabian Sea sediments awaiting discovery. 476 OTUs belonged to *Acidobacteria*, 369 OTUs to *Chloroflexi*, 283 OTUs to *Bacteroidetes*, while 182 OTUs from *Actinobacteria* and *Gemmatimonadetes* were also identified. *Firmicutes*, *Nitrospirae, Spirochaetes, Planctomycetes, Chlorobi, Fusobacteria, Tenericutes, Cyanobacteria, Verrucomicrobia, Fibrobacteres, Deinococcus-Thermus* and *Elusimicrobia* contributed less than 2% of the total identified OTUs.

## 2 Experimental design, materials, and methods

Marine sediments were collected from eastern Arabian Sea (9°59′10.9968″ N; 75° 39′ 26.4564″ E) onboard the research vessel FORV Sagar Sampada (Cruise No: 305) during August 2012 using grab at a depth of 96 m. Community DNA was isolated by modifying the classical method by utilizing liquid nitrogen for grinding sediment sample [5,6]. The V3 hypervariable region of 16S rRNA gene was amplified using 341F 5′-CCTACGGGAGGCAGCAG-3′ and 518R 5′-ATTACCGCGGCTGCTGG-3′ primer pairs with appropriate dilution of metagenomic DNA as template. Purified PCR product was used for a second PCR reaction which attached Illumina sequencing adapters and dual-index barcodes to the amplicon target. Sequencing reactions (151 bp × 2 paired end reads) were performed using the MiSeq platform (Illumina, Inc., CA, USA) following manufacturer's instructions. Raw sequencing data obtained were quantity filtered and processed using QIIME [7].
